# Spatiotemporal Beamforming: A Transparent and Unified Decoding Approach to Synchronous Visual Brain-Computer Interfacing

**DOI:** 10.3389/fnins.2017.00630

**Published:** 2017-11-15

**Authors:** Benjamin Wittevrongel, Marc M. Van Hulle

**Affiliations:** Laboratory for Neuro- and Psychophysiology, Department of Neurosciences, KU Leuven, Leuven, Belgium

**Keywords:** event-related potential, steady-state visual evoked potential, code-modulated visual evoked potential, BCI, P300, spatiotemporal filter

## Abstract

Brain-Computer Interfaces (BCIs) decode brain activity with the aim to establish a direct communication channel with an external device. Albeit they have been hailed to (re-)establish communication in persons suffering from severe motor- and/or communication disabilities, only recently BCI applications have been challenging other assistive technologies. Owing to their considerably increased performance and the advent of affordable technological solutions, BCI technology is expected to trigger a paradigm shift not only in assistive technology but also in the way we will interface with technology. However, the flipside of the quest for accuracy and speed is most evident in EEG-based visual BCI where it has led to a gamut of increasingly complex classifiers, tailored to the needs of specific stimulation paradigms and use contexts. In this contribution, we argue that spatiotemporal beamforming can serve several synchronous visual BCI paradigms. We demonstrate this for three popular visual paradigms even without attempting to optimizing their electrode sets. For each selectable target, a spatiotemporal beamformer is applied to assess whether the corresponding signal-of-interest is present in the preprocessed multichannel EEG signals. The target with the highest beamformer output is then selected by the decoder (maximum selection). In addition to this simple selection rule, we also investigated whether interactions between beamformer outputs could be employed to increase accuracy by combining the outputs for all targets into a feature vector and applying three common classification algorithms. The results show that the accuracy of spatiotemporal beamforming with maximum selection is at par with that of the classification algorithms and interactions between beamformer outputs do not further improve that accuracy.

## 1. Introduction

**Figure 1:**
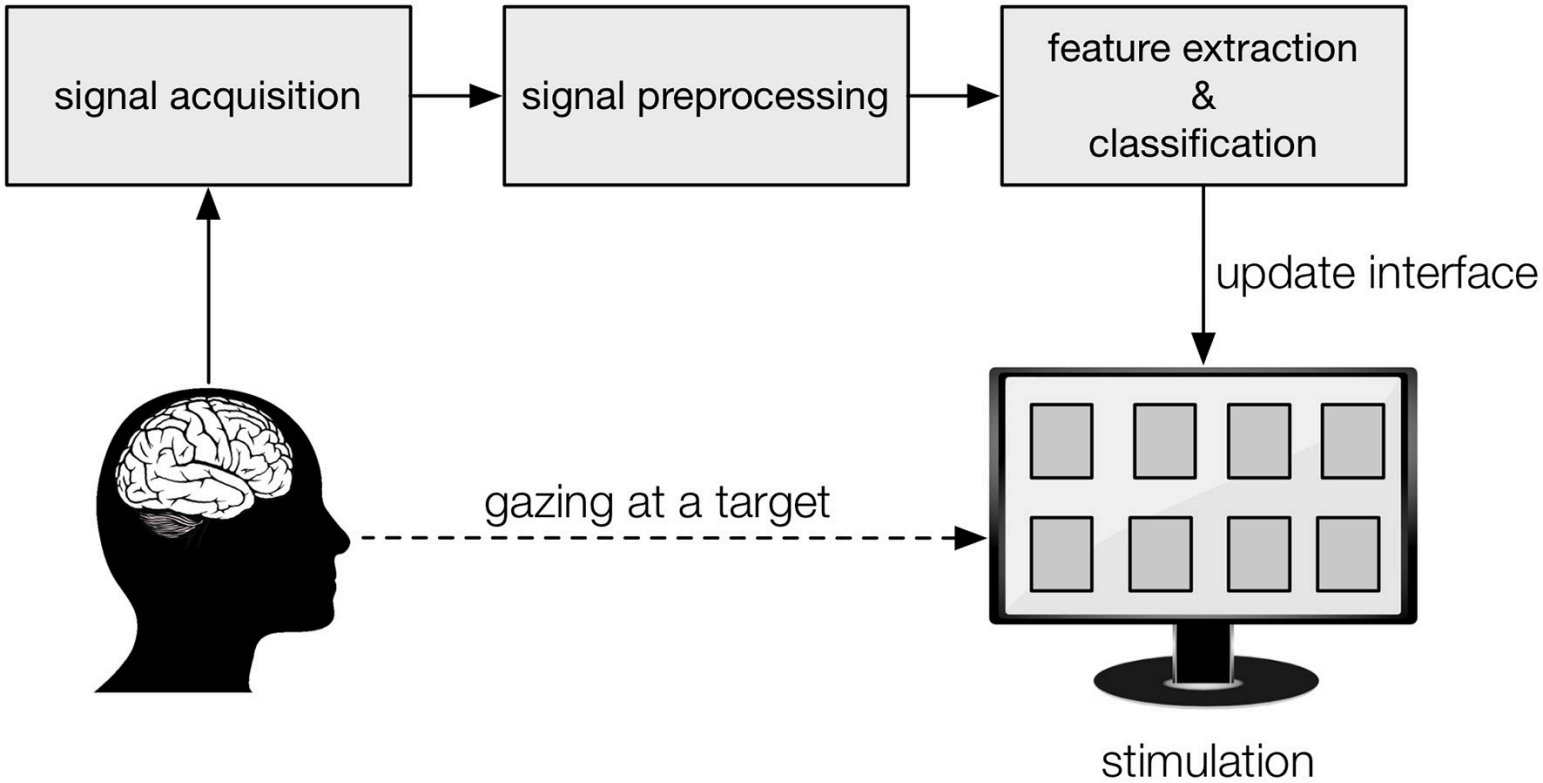
Graphical representation of a visual BCI. The subject gazes at the target on a display (“interface”), to which the desired action is associated, while his/her brain activity is being recorded. Each target is stimulated visually, in a specific manner, which leaves a trace in the recorded brain activity. From the preprocessed recordings, signal features are extracted and the classification algorithm predicts the intended target, after which the display is updated accordingly (visual feedback) and the corresponding action performed.

In a Brain-Computer Interface (BCI), signals are recorded from the brain with the aim to enable users to interact with an external device, without the need for muscular, vocal or other means of communication (Vidal, 1973). Typically, the subject is presented with a number of targets, each one representing a specific action (e.g., a letter in a spelling interface, a movement of a prosthetic limb or a wheelchair, etc.) that can be selected either by redirecting gaze or attention to the intended target (Figure 1) or by performing a mental action (e.g., imagined movement; Pfurtscheller and Neuper, 2001). Among the most performant BCI paradigms, in terms of accuracy and speed, are the ones where targets are visually stimulated and the user directs his/her gaze to target to be selected (for review, see Bin et al., 2009; Nicolas-Alonso and Gomez-Gil, 2012). Each stimulation paradigm elicits specific brain potentials, which are recorded from the scalp using electroencephalography (EEG) (Lotte et al., 2007), from the cortical surface using electrocorticography (ECoG) (Schalk and Leuthardt, 2011) or from implants such as microelectrode arrays and depth electrodes (Maynard et al., 1997; Kennedy et al., 2004). For each paradigm, analysis- and decoding techniques have been proposed tailored to maximize target prediction speed and accuracy.

One popular paradigm is based on the P300 event-related potential (ERP), an EEG component recorded over the centro-parietal region that exhibits a positive deflection in amplitude, peaking around 300 ms (whence its name), in sync with the onset of an infrequent stimulus (called oddball) to which the subject pays attention. In a P300-based BCI, the targets are overlaid with the oddball stimulus in a non-overlapping fashion (serial stimulation). The P300 can be elicited in response to a variety of sensory stimuli, including visual- (Farwell and Donchin, 1988; Sellers and Donchin, 2006; Manyakov et al., 2010a), auditory- (Sellers and Donchin, 2006; Furdea et al., 2009; Schreuder et al., 2010), and tactile stimuli (Brouwer and Van Erp, 2010). EEG recordings are typically cut into epochs, in sync with possible oddball events. However, in order to address the relatively low signal-to-noise ratio (SNR), several oddball events need to be generated and epochs averaged before P300 responses can be detected in them, resulting in a reduction in communication speed. P300-based BCIs have been most extensively studied, resulting in a wide range of techniques for EEG feature extraction and classification (Lotte et al., 2007). The most simple ones consider template matching in which a (spatio)temporal template is first learned from P300-labeled responses and then used to identify the intended target as the one with the highest correlation coefficient (Krusienski et al., 2006) or consider peak picking in which the target having the largest difference between the lowest amplitude prior to and the highest amplitude in the P300 time window is selected as winner (Farwell and Donchin, 1988). The Linear Discriminant Analysis (LDA) (Jin et al., 2013) and its variants, including Fisher LDA (Krusienski et al., 2006; Manyakov et al., 2011b), Bayesian LDA (Hoffmann et al., 2008; Manyakov et al., 2011b) and Stepwise LDA (Farwell and Donchin, 1988; Krusienski et al., 2006; Manyakov et al., 2011b), have been applied to pre-processed epochs or after feature extraction by Common Spatio-Temporal Pattern (Krusienski et al., 2007), xDAWN (Rivet et al., 2009) and linear predictive coding (Momennezhad et al., 2014). More complex methods include multilayer feed-forward neural networks (Gulcar et al., 1998; Manyakov et al., 2011b), Nuclear Norm on tensor representation (Hunyadi et al., 2013), Bayesian statistical classifiers (Pires et al., 2008), ensemble methods with SVMs and a genetic algorithm (Ghoggali et al., 2013), and a fuzzy fusion of the outcome of template matching and peak picking (Salimi Khorshidi et al., 2007; Salimi-Khorshidi et al., 2008). As far as we are aware, state-of-the-art P300 classification for BCI is achieved by the linear Support Vector Machine (SVM) (Krusienski et al., 2006; Combaz et al., 2009, 2013; Manyakov et al., 2011b) as it outperforms its non-linear (Krusienski et al., 2006; Manyakov et al., 2011b) counterparts. The recently introduced spatiotemporal beamforming filter has been shown to be at par with an optimized linear SVM for P300 detection (Wittevrongel and Van Hulle, 2016a).

Unlike the P300 paradigm, which is inherently serial in nature, the SSVEP paradigm encodes the targets using periodically and simultaneously flickering stimuli, and in which each target adopts a unique frequency (Regan, 1979; Middendorf et al., 2000), unique phase (Lee et al., 2010; Lopez-Gordo et al., 2010; Manyakov et al., 2012) or unique frequency-phase combination (Jia et al., 2011). EEG signals are recorded over the occipital cortex and are expected to carry the same signal characteristics as the target being gazed at. Hence, SSVEP decoding methods are traditional relying on frequency domain methods such as the Fourier transform (Gao et al., 2003) and the Continuous Wavelet Transform (Zhang et al., 2010). Other frequency-detection algorithms include the Minimum Energy Combination (Friman et al., 2007; Chumerin et al., 2013; Combaz et al., 2013), the Multivariate Synchronization Index (MSI) (Zhang et al., 2014a, 2017) and time-domain analysis (Luo and Sullivan, 2010; Manyakov et al., 2010b). Among the machine learning algorithms that have been used for SSVEP target identification are Bayesian LDA (Cecotti, 2010), SVM (Singla and Haseena, 2014), k-Nearest Neighbor (kNN) (Kwak et al., 2015), and several neural network architectures (Manyakov et al., 2011a, 2013; Singla and Haseena, 2014; Kwak et al., 2017). However, the most accurate SSVEP decoding methods are based on the Canonical Correlation Analysis (CCA) (Lin et al., 2007), and its numerous extensions (Pan et al., 2011; Zhang et al., 2011; Chen et al., 2014, 2015; Nakanishi et al., 2014; Zhang et al., 2014b; Vu et al., 2016), and spatiotemporal beamforming (Wittevrongel and Van Hulle, 2016b,c).

Whereas the P300 paradigm encodes targets in terms of ERP responses and SSVEP in terms of frequency and/or phase combinations, the code-modulated VEP (cVEP) paradigm encodes the targets using lagged versions of a binary coding sequence. While record-breaking communication speed was achieved when it was introduced, it has been considerably less studied (Gao et al., 2014), and fewer decoding algorithms have been proposed for this paradigm. Originally, template matching (Bin et al., 2009) was used to identify the gazed target from averaged epochs, but also the CCA algorithm has been applied (Bin et al., 2011; Aminaka et al., 2015a; Wei et al., 2016) as well as SVMs (Aminaka et al., 2015b,c) and spatiotemporal beamforming (Wittevrongel et al., 2017). The latter two have shown state-of-the-art performance with shorter stimulation times.

Importantly, what the majority of the reported decoding algorithms have in common is that they have been construed to maximize performance of a given paradigm, even when used in a particular context. We argue that one of these algorithms, the spatiotemporal beamformer, can achieve state-of-the-art performance in all three paradigms. The beamformer is a filter that estimates the contribution of an a-priori defined activation pattern (i.e., a template, a signal-of-interest) into new data. It can appear as a spatial, temporal or spatiotemporal filter. Unlike in our previous work (Wittevrongel and Van Hulle, 2016a,b; Wittevrongel et al., 2017), which was aimed at investigating the beamformer in the context of a specific paradigm, the first goal of this manuscript is to describe a generalized and unified approach to spatiotemporal beamforming for decoding synchronous EEG responses by demonstrating its versatility in the context of synchronous visual BCI. Secondly, in addition to a simple maximum-beamformer output classification, we also investigate whether interactions between beamformer outputs can increase decoder accuracies. We end by indicating future directions for spatiotemporal beamforming in BCI.

## 2. Methods

### 2.1. Data acquisition and processing

The data used in this study has been collected in previous offline BCI studies. For more details about the experimental setting, interface and recordings, we refer the reader to the corresponding publications. All three studies were carried out in accordance with the recommendations and approval of the ethics committee of our university hospital UZ Leuven with written informed consent from all subjects. All subjects gave written informed consent in accordance with the Declaration of Helsinki.

Data for the P300 paradigm was collected using an interface consisting of 9 targets (Wittevrongel and Van Hulle, 2016a). In each trial, subjects were asked to direct their gaze to a cued target and continue to focus as all targets were individually highlighted 15 times for 100 ms (with 100 ms inter-stimulus interval) in pseudorandom order. In response to the cued target, when highlighted, a P300 response will be elicited. All targets were used as cue 4 times, leading to a total of 36 trials, each consisting of 15 “P300 epochs” (i.e., where the cued target was highlighted) and 120 epochs where another target was highlighted. Data from 21 subjects was collected using an ActiveTwo system (BioSemi, The Netherlands) with 32 active Ag/AgCl electrodes (and 4 additional electrodes around the eyes) at a sampling rate of 2,048 Hz. The raw data was offline referenced from the Common-Mode Sense (CMS) reference to average of the mastoid signals, corrected for eye artifacts using the RAAA-method (Croft and Barry, 2000), filtered between 0.5 and 15 Hz and cut in 0.6-s epochs time-locked to the onset of the stimulation. Each epoch was baselined by subtracting the average of the 100 ms pre-onset activity and downsampled to 64 Hz.

SSVEP data was obtained from an interface consisting of 4 targets, each adopting an unique frequency-phase combination of 12 or 15 Hz and 0 or π radians (Wittevrongel and Van Hulle, 2016b). During a trial, subject were asked to direct their gaze to a cued target and continue to focus when all targets were simultaneously flickering at their unique frequency-phase combinations for 5 s. All targets were cued 15 times, leading to a total of 60 5-s trials. Data from 20 subjects was recorded using an ActiveTwo system (BioSemi, The Netherlands) with 32 active Ag/AgCl electrodes (and 4 additional electrodes around the eyes) at a sampling rate of 2048 Hz. The raw data was offline referenced from the CMS reference to the average of the mastoid signals, corrected for eye artifacts using the RAAA-method (Croft and Barry, 2000), filtered between 4 and 20 Hz, cut in 5-s trials time-locked to the onset of the flickering, and downsampled to 512 Hz.

For the cVEP study, a 32-target interface was used (Wittevrongel et al., 2017). Targets were encoded using lagged versions of a binary m-sequence of length 63, presented at a stimulation rate of 120 Hz (1 m-sequence = 0.525 s). Each trial started with a cue, to which the subjects need to direct their gaze and to continue focusing while all targets were simultaneously presented with their unique lagged m-sequences. The sequences were repeated 10 times. Each target was cued 5 times, leading to a total of 160 trials. Data from 17 subjects was recorded using a Neuroscan SynampsRT device (Compumedics, Australia) with 32 active Ag/AgCl electrodes at a sampling rate of 1,000 Hz with ground and reference electrodes at FPz and AFz, respectively. The data was offline referenced to the average of the mastoid signals, filtered between 4 and 31 Hz, cut into trials that are time-locked to the onset of the stimulation, and finally downsampled to 120 Hz. Since no ocular electrodes were included in the recording, the data was not corrected for eye artifacts.

### 2.2. Spatiotemporal beamforming

The beamforming principle was originally introduced as a spatial filter for radar, sonar and seismic data analysis (Van Veen and Buckley, 1988), but has also been employed in EEG analysis to isolate the signal originating from a predefined brain location (Van Veen et al., 1997), for ERP analysis (Treder et al., 2016), and to build a BCI application based on imagined movement detection (Grosse-Wentrup et al., 2009).

van Vliet et al. (2015) introduced a spatiotemporal extension to beamforming for single-trial N400 ERP detection in the context of processing semantic stimuli. To calculate the spatiotemporal filter, a spatiotemporal activation pattern **A** ∈ ℝ^*m*×*n*^ needs to be defined, where *m* represents the number of channels (here, EEG electrodes) and *n* the number of samples. This activation pattern represents the signal-of-interest (or template) and can be obtained a-priori (van Vliet et al., 2015) or based on actual training data (Wittevrongel and Van Hulle, 2016a,b; Wittevrongel et al., 2017). The resulting beamformer **w** ∈ ℝ^1×*mn*^ is a multivariate filter that optimally isolates the targeted response from noise and possible other unrelated activity, by taking into account the information contained in the covariance matrix **Σ** ∈ ℝ^(*mn*)×(*mn*)^ estimated from the available (training) data.

The original formulation of the Linearly Constrained Minimum Variance (LCMV) spatial beamformer ${\text{w}}_{sp}\in {\mathbb{R}}^{m\times 1}$ minimizes the variance of the beamformer output ${\text{w}}_{sp}^{⊺}\text{S}$:

(1) $$\underset{w_{sp}}{\text{arg min}}w_{sp}^{⊺}S{\left(w_{sp}^{⊺}S\right)}^{⊺}\Rightarrow \underset{w_{sp}}{\text{arg min}}w_{sp}^{⊺}\Sigma_{sp}w_{sp},$$

where $\Sigma_{sp}\in {\mathbb{R}}^{m\times m}$ is the spatial covariance matrix of an EEG trial **S** ∈ ℝ^*m*×*n*^. By adding the linear constraint:

(2) $$a_{sp}^{⊺}w_{sp}=1,$$

where ${\text{a}}_{sp}\in {\mathbb{R}}^{m\times 1}$ is the spatial activation pattern, trivial solutions of (1) are avoided, and signals that are similar to **a**_*sp*_ will be mapped to a value close to 1, allowing for an easy measure of similarity. The solution of (1) under constraint (2) can be found using the method of Lagrange multipliers (Van Veen et al., 1997):

(3) $$w_{sp}=\frac{\Sigma_{sp}^{-1}a_{sp}}{a_{sp}^{⊺}\Sigma_{sp}^{-1}a_{sp}}.$$

The spatial beamformer can be expanded to a spatiotemporal variant as follows. Let *S* ∈ ℝ^*m*×*n*×*r*^ be *r* trials, **X** ∈ ℝ^*r*×(*mn*)^ a matrix where each row *l* is obtained by concatenating the rows of a corresponding trial *S*[*, *, *l*] with *l* ∈ [1..*r*], **Σ** ∈ ℝ^(*mn*)×(*mn*)^ the covariance matrix of **X**^⊺^, and **a**^⊺^ ∈ ℝ^1×(*mn*)^ a vector containing the concatenated rows of the spatiotemporal activation pattern **A**. The spatiotemporal LCMV beamformer **w** ∈ ℝ^(*mn*)×1^ with the linear constraint **a**^⊺^**w** = 1 can now be calculated as:

(4) $$w=\frac{\Sigma^{-1}a}{a^{⊺}\Sigma^{-1}a},$$

and applied to the data as a simple weighted sum:

(5) y=sw,

where **s** ∈ ℝ^1×(*mn*)^ indicates the concatenated rows of a segment **S** and *y* represents the contribution of the activation pattern **A** in **S**.

### 2.3. Beamformer construction and feature extraction

**Figure 2:**
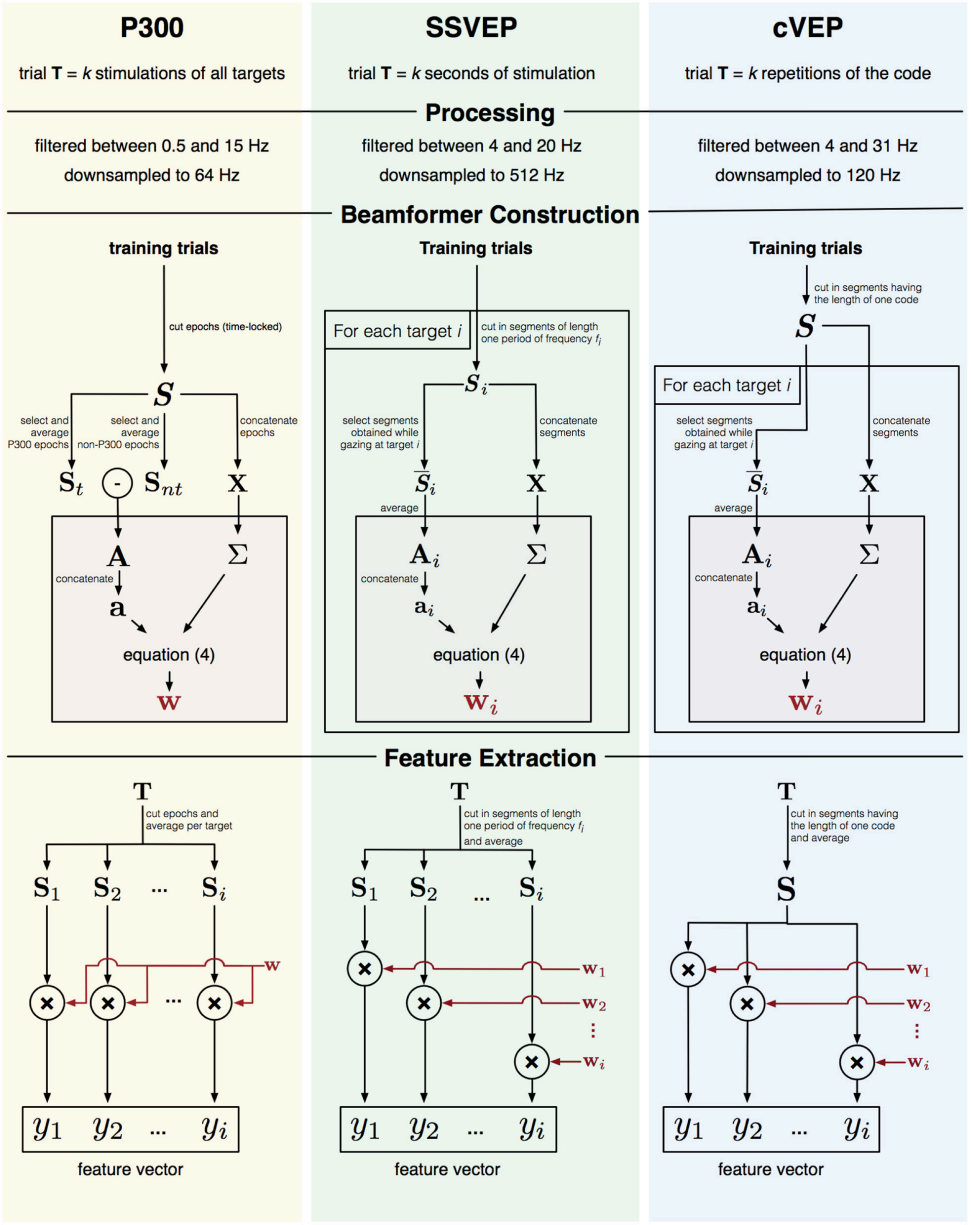
Graphical representation of feature extraction and beamformer construction for three paradigms.

As the activation pattern captures the signal-of-interest, each paradigm (P300, SSVEP, cVEP) will have unique activation patterns, and since EEG responses might different across subjects, beamformers should be constructed for each subject individually (Wittevrongel and Van Hulle, 2016a). In the next few sections, we will describe for each paradigm how one or more beamformers (BF) are constructed and used to extract feature vectors. A visual depiction of this process is given in Figure 2.

#### 2.3.1. P300

For a P300-based BCI the activation pattern **A** represents the P300 ERP. One trial **T** in the P300 paradigm consists of *k* stimulations (i.e., highlights) of all targets. To obtain the activation pattern **A**, all training trials are first cut into time-locked epochs *S* from 0 to 600 ms post-stimulus onset and their baseline subtracted. The activation pattern **A** can then be obtained as the difference between the average (**S**_*t*_) of the P300 epochs (i.e., when the target being gazed at is highlighted) and the average (**S**_*nt*_) of the non-P300 epochs. The covariance matrix **Σ** is calculated from *S* (using the concatenation approach described above), and a single beamformer **w** constructed.

To obtain the feature vector for a trial **T**, the trial is first cut into time-locked epochs and all epochs corresponding to the same target are averaged. Each averaged epoch ${\text{S}}_{i}\in {\mathbb{R}}^{m\times n}$ is then filtered by the beamformer **w** to obtain an value *y*_*i*_, which represents the P300 contribution for target *i*.

#### 2.3.2. SSVEP

Targets in the SSVEP paradigm are encoded with unique frequency-phase combinations, and one trial **T** consists of *k* seconds of SSVEP stimulation, during which all targets are simultaneously flickering at their assigned frequency-phase combinations. Unlike for the P300 case, each target elicits a specific frequency and phase response, and it is therefore not possible to define a single activation pattern to discriminate targets, but rather multiple activation patterns, each one tailored to one target, are needed. To capture the frequency-phase response to target *i*, a time-domain analysis (Luo and Sullivan, 2010; Manyakov et al., 2010b) is adopted: each training trial **T** is cut into consecutive, non-overlapping segments **S**_*i*_ with a length *n* equal to a single period of stimulus frequency *f*_*i*_. The activation pattern **A**_*i*_ for target *i* is then given by averaging the segments (${\bar{\text{S}}}_{i}\subset {\text{S}}_{i}$) extracted from the trials during which target *i* was cued. The covariance matrix **Σ** was calculated from ***S***_*i*_ and beamformer **w**_*i*_ from Equation (4).

The feature vector for a trial **T** contains the beamformer outputs *y*_*i*_ for each target *i*. To obtain *y*_*i*_, the trial is cut into segments of length one period of frequency *f*_*i*_, the segments are averaged and filtered using the corresponding beamformer **w**_*i*_.

To improve classification accuracy, the first 120 ms of each trial were removed prior to cutting the segments, as the SSVEP response is not stable during this time. (Nakanishi et al., 2014; Wittevrongel and Van Hulle, 2016b).

#### 2.3.3. cVEP

In the cVEP paradigm, targets were encoded using lagged versions of a binary m-sequence, which was repeated *k* times over the course of one trial **T**. Similar to the SSVEP paradigm, each target elicited a unique response and several activation patterns (one for each target) were defined. Each training trial **T** was cut into a maximal number of 0.525-s consecutive, non-overlapping segments ***S***. Note that the 0.525 s corresponds to the time needed to display one full m-sequence. The activation pattern **A**_*i*_ for target *i* was then obtained as the average of the segments ${\bar{\text{S}}}_{i}\subset \text{S}$ in response to target *i*. As for SSVEP, the covariance matrix **Σ** was calculated from *S*, and beamformer **w**_*i*_ from Equation (4).

The feature vector for a trial is constructed by cutting the trial into segments (using the same method as above), averaging the segments (**S**) and independently applying the beamformer **w**_*i*_ to obtain the corresponding *y*_*i*_ for each target *i*.

### 2.4. Target identification

**Figure 3:**
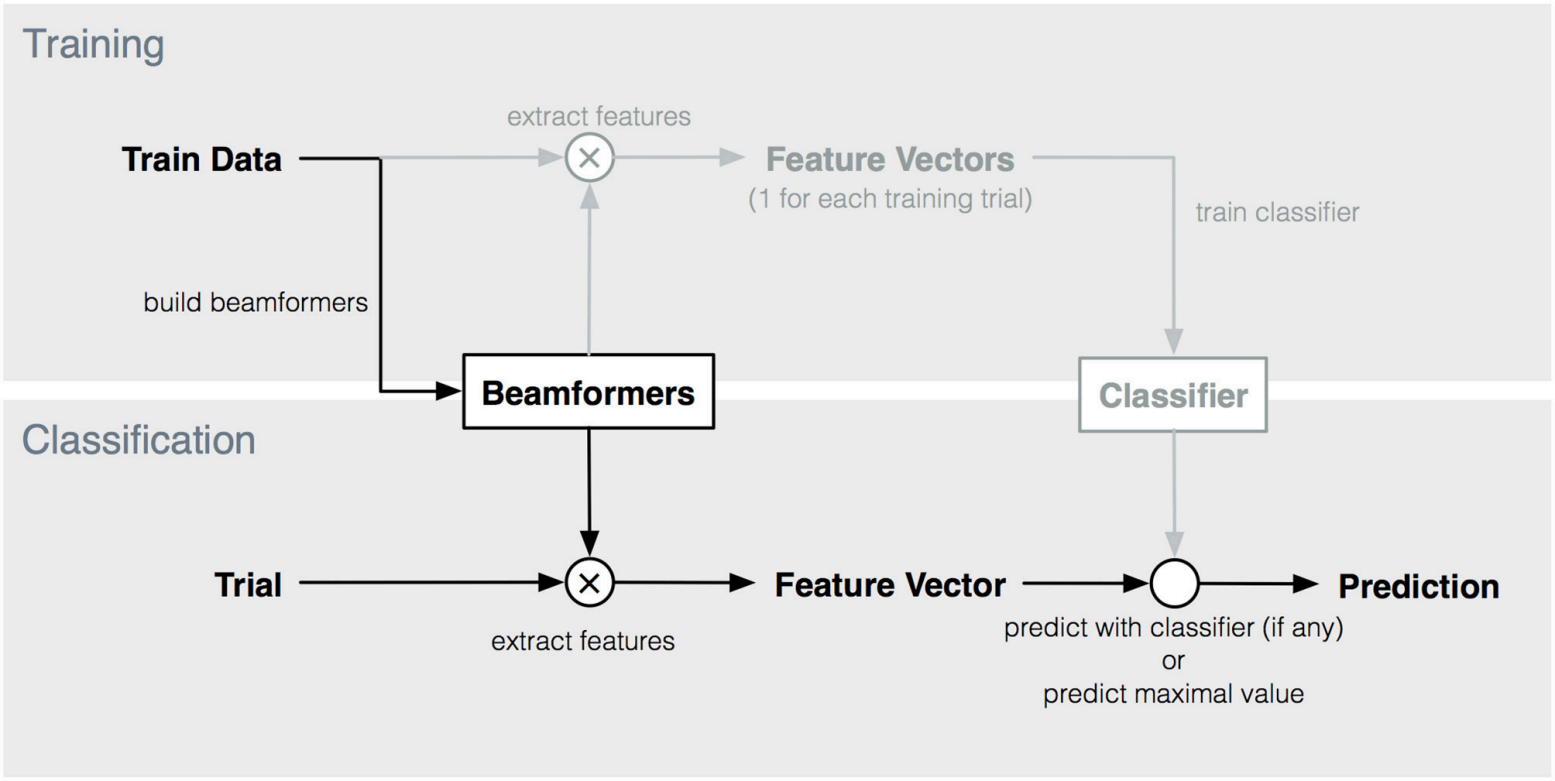
Overview of training and classification procedure. Elements indicated in lighter color are not used in maximum-based target prediction.

Based on the feature vector, a prediction for the intended target was made. We compared several prediction strategies. As the feature vectors contain estimates of the degree to which the activation pattern of each target is present in the trial, the most naive prediction is to select the target with the highest score (i.e., prediction = max(*y*_*i*_)). However, as alternatives, we also applied three common classifiers (Nearest Neighbor (NN), LDA and SVM) to the features vectors. These classifiers can take into account possible interactions between beamformer outputs of different targets (i.e., features in the feature vector). The feature vectors of the training trials were used to train these classifiers. The complete procedure is depicted in Figure 3. The three classifiers were implemented by using the corresponding built-in functions in Matlab (2015) (*fitcdiscr, fitcknn, fitcecoc*).

### 2.5. Performance estimation

Classification accuracy was estimated using cross-validation (4-fold for the P300 data and 5-fold for SSVEP and cVEP data), for increasing signal lengths (i.e., stimulus repetitions for P300 and cVEP and stimulation length for SSVEP).

**Figure 4:**
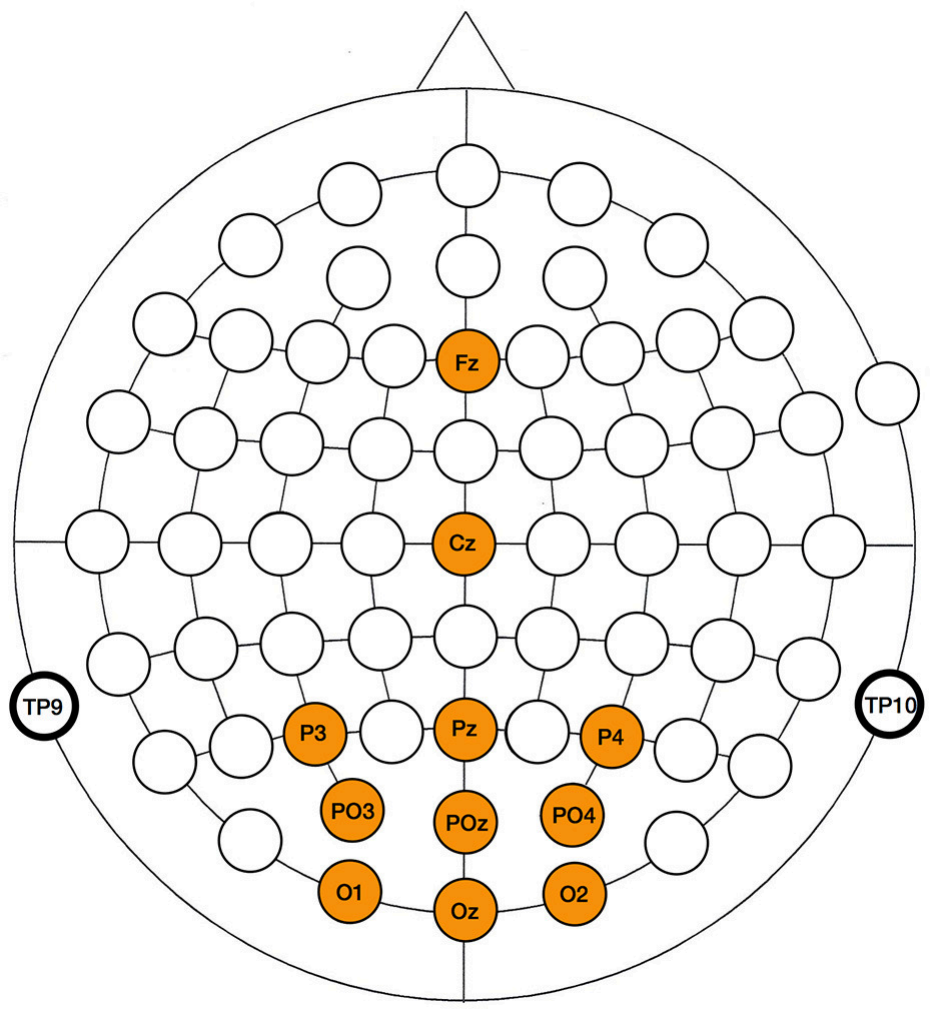
Scalp locations of electrodes used in the EEG analysis of the three paradigms.

For all paradigms, the same electrode set was included in the analysis, consisting of *Fz, Cz, Pz, Oz, O1, O2, PO3, PO4, P3*, and *P4*. The locations of the included electrodes (Figure 4) cover the scalp areas where routinely P300 (centro-parietal), SSVEP (occipital) and cVEP (parieto-occipital) responses are detected. Note that we do not attempt to optimize the electrode sets of the paradigms, but rather consider the same assembly for all three paradigms, which adds to the genericness of our approach.

### 2.6. Statistics

Since the distributions do not consistently follow a Gaussian distribution, we adopted the non-parametric (two-tailed) Wilcoxon signed rank test. We used this test to compare the accuracies of the different classifiers. The significance threshold was set to 0.0083 (=$\frac{0.05}{6}$) after applying the Bonferroni correction for multiple comparisons.

### 2.7. Data availability

All data and analysis scripts are available at: https://kuleuven.box.com/v/SpatiotemporalBeamforming.

## 3. Results

**Figure 5:**
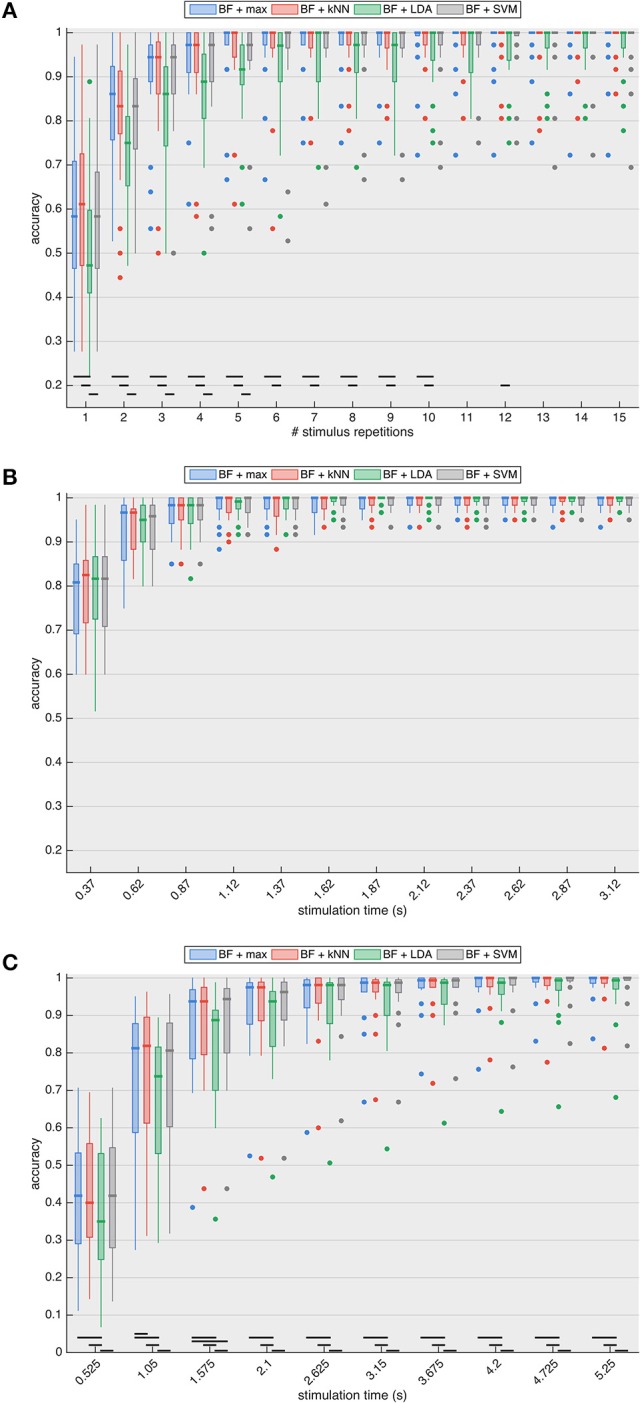
Accuracy of target identification for the **(A)** P300, **(B)** SSVEP, and **(C)** cVEP paradigms with increasing stimulation lengths, and with target prediction based on the maximal beamformer (BF + max) output, as well as the prediction based on the k-Nearest Neighbor (kNN), Linear Discriminant Analysis (LDA) and Support Vector Machine (SVM) algorithms, using the beamformer outputs of all targets as feature vector. The results show that classification algorithms do not outperform maximum-based prediction, indicating that interactions between beamformer outputs do not increase target prediction accuracy. The black horizontal lines indicate significant differences based on the paired two-tailed Wilcoxon signed rank test with Bonferroni correction. Accuracies of all subjects are summarized using boxplots: the thick horizontal line indicates the median accuracy, the box stretches from the 1st to the 3rd quartile, the lines extending from the box indicate the minimum and maximum value within 1.5 times the interquartile range from the 1st and 3rd quartile, respectively, and the dots represent outliers.

Figure 5A shows the classification accuracies of the four classifiers for the P300 paradigm with increasing stimulus repetitions (1 repetition = 1.8 s). For all stimulus repetitions in the P300 case, there is no significant difference between maximal beamformer output prediction and NN- or SVM-based classification. LDA-based prediction consistently has the lowest median accuracy and is significantly different up to 5 (compared to SVM, *p* < 0.003) and 10 (compared to max and NN, *p* < 0.008) stimulus repetitions. All four prediction approaches require at least two stimulus repetitions to surpass the 70% accuracy threshold (median accuracy of 86.1% for maximum-, 83.3% for kNN- and SVM- and 75.0% for LDA-based prediction) deemed necessary for establishing reliable communication (Kübler et al., 2004; Kübler and Birbaumer, 2008; Brunner et al., 2011; Combaz et al., 2013).

With the SSVEP paradigm (Figure 5B), the target identification accuracies of the four classification approaches are not significantly different for any of the stimulation lengths. The accuracies of all classifiers increase considerably up until 1.12 s stimulation, after which the maximal median accuracy of 100% is reached (for maximum-, kNN- and SVM-based prediction) and remains consistent for longer stimulation lengths. Note that the interquartile ranges (as well as the number of outliers) are considerably smaller compared to the P300 paradigm, indicating a higher consistency across subjects, which may be due to the fact that SSVEP is an automatic visual (sensory) response while P300 is a cognitive potential and thus more influenced by ongoing mental activity (e.g., attention).

Similar to the P300-case, for the cVEP paradigm (Figure 5C), LDA-based prediction consistently exhibits the lowest median accuracy for all signal lengths. While the maximal-based prediction is significantly different from NN for a stimulation length of 1.05 s (*p* = 0.006, two-tailed Wilcoxon signed rank test) and from SVM for 1.625 s stimulation (*p* = 0.002, two-tailed Wilcoxon signed rank test), maximal-, NN- and SVM-based predictions are not significantly different for any other stimulation lengths. Two repetitions of the m-sequence (i.e., stimulation length of 1.05 s) are necessary for the median to surpass the 70% accuracy threshold (median accuracy of 81.3% for maximum-, 81.9% for kNN-, 80.6% SVM-, and 73.7% for LDA-based prediction). For all classifiers, the accuracies steadily increase with increasing stimulation length.

## 4. Discussion

In this manuscript, we described a transparent and unified decoding approach to synchronous visual BCI, and showed its feasibility for three popular paradigms: P300, SSVEP, and cVEP. The feature vector of a trial was determined as the output of spatiotemporal beamformer(s), and we compared four methods for predicting the intended target.

The accuracies obtained by the NN- and SVM-based predictions are, with two exceptions for the cVEP paradigm, not significantly different from maximum-based prediction. The additional information given by the interaction between the beamformer outputs is not beneficial for target identification, and is even detrimental when adopting an LDA-based classifier. Furthermore, maximum-based prediction has a computational advantage as no feature extraction and classifier training is required after building the spatiotemporal beamformer(s) (see Figure 3).

For all three tested paradigms, an identical electrode set was included in the analysis to demonstrate the versatility and robustness of the spatiotemporal beamforming approach. As the aim of this study was to describe a unified methodology for synchronous BCI, the accuracies reported in this manuscript are likely not optimal and could be improved by optimizing the electrode set (Lal et al., 2004; Schröder et al., 2005; Lv and Liu, 2008; Arvaneh et al., 2011; Barachant and Bonnet, 2011; Wittevrongel et al., 2017) or the filtering range (Song and Epps, 2007; Manyakov et al., 2010a) for each subject individually. In our previous work (Wittevrongel and Van Hulle, 2016a,b; Wittevrongel et al., 2017), we have shown that the accuracies obtained by the spatiotemporal beamforming approach are at par of even outperform the state-of-the-art classifiers of the respective paradigms, and that training beamformers is often faster than alternative classification methods such as SVM, which would allow experimenters to explore the aforementioned optimizations within a reasonable amount of time.

Both spatial and spatiotemporal filtering serve to improve the SNR, which allows for more accurate ERP analysis or classification performance. While the LCMV beamformer approach with a data-driven activation pattern has also been applied to spatial filtering (Treder et al., 2016), the spatiotemporal filter used in this study has the advantage to jointly model both the spatial and temporal characteristics of the signal-of-interest. For the P300 ERP, for example, it has been shown that target prediction is more accurate when the early visual ERP components (e.g., N200 elicited over the occipital cortex) are included in the analysis (Bianchi et al., 2010; Kaufmann et al., 2011a), and many studies have therefore investigated stimulation paradigms that elicit additional ERP components (Kaufmann et al., 2011b; Jin et al., 2012, 2015). A spatial filter tuned to the centro-parietal P300 component would filter out these additional components, causing a reduction in prediction accuracy.

In recent years, various complex algorithms have been proposed that often rely on (multiple) extensions of an existing algorithm and that are tailored to a specific paradigm (e.g., the extensions for MSI Zhang et al., 2017 and CCA Lin et al., 2007; Pan et al., 2011; Zhang et al., 2011; Chen et al., 2014, 2015; Nakanishi et al., 2014; Zhang et al., 2014b; Vu et al., 2016 for SSVEP detection). Other proposed approaches apply a black-box classification algorithm (SVM, Neural Networks), where the experimenter does not have control over the pattern on which target discrimination is based. However, in the context of BCI, this pattern is in most cases well known, and in previous work we have shown that for example the pattern learned by a state-of-the-art black-box SVM in the context of a P300-BCI is very similar to the activation pattern obtained by simple averaging such as in the spatiotemporal beamformer approach (Wittevrongel and Van Hulle, 2016a). Unlike these algorithms, the beamforming approach described in this manuscript is less complex (as desired, cfr. Occam's razor), fully transparent and applicable to various synchronous stimulation paradigms.

In future work, we will further develop the spatiotemporal beamformer. At this point, the main bottleneck is to estimate the high-dimensional covariance matrix, which, to be accurate, requires a significant amount of training data (cfr. the curse of dimensionality Pruzek, 1994; Schoukens and Pintelon, 2014). Compared to the spatial beamformer, the covariance matrices in the spatiotemporal variant consider the complete temporal dimension of the signal-of-interest for each evaluated channel (hence its dimension of (*mn*)×(*mn*), with *m* the number of channel and *n* the number of (time) samples). For the 10 channels selected in our study, the covariance matrices had the following dimensions: 380 (= 10 channels × 38 samples) for P300; 420 or 340 (depending on the frequency) for SSVEP; and 630 for cVEP. Furthermore, since our approach requires spatiotemporal samples, training data is considerably harder to obtain. For a spatial beamformer, one typically can collect hundreds of samples in one second, while for the spatiotemporal variant the number of samples that can be collected per second depends on the temporal characteristics of the signal-of-interest. In our study, the P300 epochs were 0.6 s long (albeit that they are partially overlapping), SSVEP segments required 1/12 or 1/15 s of stimulation (depending on the frequency), and one signal-of-interest in the cVEP paradigm was 0.525 seconds long, leading to a mere 3 (= 0.6 s with 0.2 s stimulus onset asynchrony), 12 or 15, and 1.9 samples extracted from the first second of stimulation for P300, SSVEP, and cVEP, respectively. In addition to reducing the number of channels included, alternative methods for the estimation of the covariance matrix can be considered. In this and previous studies, we estimated a pooled covariance matrix by including non-targeted segments for the covariance matrix estimation, which is often used in cases when the amount of samples is smaller than the dimensions of the covariance matrix (e.g., facial recognition, Edwards et al., 1998, BCI, Vidaurre and Blankertz, 2010). However, several methods for covariance matrix estimation with a small number of samples have been described (e.g., shrinkage, Ledoit and Wolf, 2004, regularization, Friedman, 1989, among others) and can be assessed in terms of improving the estimation of the covariance matrix.

At this time, we applied the beamformer for synchronous signals, but it can likely be extended for asynchronous detection of spatiotemporal signals of interest. As the beamformer outputs can be interpreted, the spatiotemporal beamformer in combination with a sliding window could be used as transformation of multi-electrode EEG to a single-trace estimate of the contribution of a spatiotemporal template in ongoing EEG recordings. An important aspect of this development will be to limit to amount of false positive detections.

## 5. Conclusion

In this study, we described spatiotemporal beamforming as a unified approach for target identification in BCI applications that rely on EEG responses synchronized to the stimulation onset. We have shown the versatility of our approach by applying it to three popular visual stimulation paradigms (P300, SSVEP, and cVEP), and demonstrated its robustness since high accuracies were reached with an identical (thus, non-optimized) electrode set for all paradigms. Unlike other classification algorithms, the spatiotemporal beamformer is fairly straightforward and fully transparent as the experimenter has full control over the activation pattern used for target discrimination. Finally, we showed that selecting the target with the highest beamformer output suffices to achieve a competitive accuracy, and that by accounting for possible interactions between beamformer outputs, accuracy does not further improve.

## Author contributions

BW conducted the analysis. Both authors wrote and reviewed the manuscript.

### Conflict of interest statement

The authors declare that the research was conducted in the absence of any commercial or financial relationships that could be construed as a potential conflict of interest.
